# NOX2-Induced Activation of Arginase and Diabetes-Induced Retinal Endothelial Cell Senescence

**DOI:** 10.3390/antiox6020043

**Published:** 2017-06-15

**Authors:** Modesto Rojas, Tahira Lemtalsi, Haroldo A. Toque, Zhimin Xu, David Fulton, Robert William Caldwell, Ruth B. Caldwell

**Affiliations:** 1 Vascular Biology Center, Augusta University, 1459 Laney Walker Boulevard, Augusta, GA 30912-2500, USA; mrojas@augusta.edu (M.R.); tlemtalsi@augusta.edu (T.L.); zhxu@augusta.edu (Z.X.); dfulton@augusta.edu (D.F.); 2 VA Medical Center, One Freedom Way, Augusta, GA 30904-6285, USA; 3 Department of Pharmacology & Toxicology, Augusta University, 1459 Laney Walker, Boulevard, Augusta, GA 30912-2500, USA; hflorestoque@augusta.edu (H.A.T.); wcaldwel@augusta.edu (R.W.C.)

**Keywords:** diabetic retinopathy, retina, endothelial cell, oxidative stress, NOX2/NADPH oxidase 2, NO, NOS, arginase 1, senescence associated β-galactosidase

## Abstract

Increases in reactive oxygen species (ROS) and decreases in nitric oxide (NO) have been linked to vascular dysfunction during diabetic retinopathy (DR). Diabetes can reduce NO by increasing ROS and by increasing activity of arginase, which competes with nitric oxide synthase (NOS) for their commons substrate l-arginine. Increased ROS and decreased NO can cause premature endothelial cell (EC) senescence leading to defective vascular repair. We have previously demonstrated the involvement of NADPH oxidase 2 (NOX2)-derived ROS, decreased NO and overactive arginase in DR. Here, we investigated their impact on diabetes-induced EC senescence. Studies using diabetic mice and retinal ECs treated with high glucose or H_2_O_2_ showed that increases in ROS formation, elevated arginase expression and activity, and decreased NO formation led to premature EC senescence. NOX2 blockade or arginase inhibition prevented these effects. EC senescence was also increased by inhibition of NOS activity and this was prevented by treatment with a NO donor. These results indicate that diabetes/high glucose-induced activation of arginase and decreases in NO bioavailability accelerate EC senescence. NOX2-generated ROS contribute importantly to this process. Blockade of NOX2 or arginase represents a strategy to prevent diabetes-induced premature EC senescence by preserving NO bioavailability.

## 1. Introduction 

Diabetic retinopathy (DR) is a major cause of vision loss and blindness in working age adults [1]. DR affects all retinal cells, especially the microvasculature [2,3]. Vision loss can occur due to macular edema, proliferative neovascularization or retinal neurodegeneration [4,5]. Early microvascular changes in the diabetic retina, including leukostasis and increased vascular permeability, have been linked to increased formation of reactive oxygen species (ROS) [6,7]. Studies in a variety of tissues have shown that proper vascular function depends on appropriate basal production of nitric oxide (NO) and that NO function as an endothelial-derived vasodilator and anti-inflammatory factor is compromised in diabetes [8]. Diabetes can decrease NO bioavailability by several mechanisms, including increased production of superoxide. Superoxide will react rapidly with NO to form peroxynitrite. Peroxynitrite and other oxidants can cause NOS uncoupling due to oxidation of the NOS co-factor BH_4_ and/or by increasing expression/activity of the ureohydrolase enzyme arginase [9]. Excessive arginase activity can also cause uncoupling of endothelial NOS (eNOS) due to depletion of their common substrate l-arginine [10]. Uncoupled eNOS forms less NO and more O_2_^−^ leading to further increases in oxidative stress. A reduction in NO and increase in ROS also causes endothelial cell (EC) activation, which leads to alterations in gene expression and increased secretion of pro-inflammatory cytokines [11,12]. Similar changes have been detected in cells with senescence-associated secretory phenotype (SASP) [13]. Although cells in senescence are metabolically active, their physiological functions are limited. Studies with human umbilical vein endothelial cells (HUVEC) exposed to intermittent high glucose conditions have demonstrated the role of NADPH oxidase-derived ROS in the development of SASP [14]. Overexpression of either arginase isoform has been reported to promote senescence of HUVEC [15,16]. An association between diabetes and the development of SASP in retinal cells has also been reported [17,18]. However, the underlying mechanisms are not yet understood.

Our previous studies in diabetic mice have shown that NOX2/NADPH oxidase-derived ROS have an important role in diabetes-induced retinal inflammation and early signs of retinopathy [19,20,21]. Diabetes-induced increases in ROS production, vascular endothelial growth factor (VEGF) and intercellular adhesion molecule 1 (ICAM1) expression, leukocyte adhesion to the retinal vessels, and breakdown of the blood-retinal barrier were all prevented by deletion of the NADPH oxidase 2 (NOX2) gene [19,21]. Our studies have shown that knockdown of arginase 1 also prevents the diabetes-induced increases in ROS formation while preserving NO bioavailability and limiting vascular inflammation [22]. Decreases in NO bioavailability have been linked to SASP in aorta of the Zucker rat model of metabolic syndrome [23]. Furthermore, increases in ROS have been shown to reduce NO formation by a mechanism involving increases in arginase expression/activity and uncoupling of NOS [24,25]. Thus, we hypothesized that diabetes-induced activation of NOX2/NADPH oxidase promotes the development of SASP by mechanisms involving ROS-induced increases in arginase expression/activity and decreases in bioavailable NO. Experiments using animal and tissue culture models confirmed this hypothesis. The data show that increases in NOX2 or arginase activity act at different levels in the same pathway but with the same end results. Inhibition or blockade of NOX2 or arginase normalizes ROS production and preserves NO levels while limiting SASP. 

## 2. Materials and Methods

### 2.1. Mouse Model 

Studies using mice were conducted according to the recommendations in the National Institutes of Health Guide for the Care and Use of Laboratory Animals. The Animal Protocol was approved by the Institutional Animal Care and Use Committee of Augusta University (2008-0243). All surgery was performed under anesthesia (Ketamine/Xylazine). Mice were rendered diabetic by injections of streptozotocin (STZ, 75 mg/ kg, i.p on alternate days for 3 days). Diabetes was confirmed by blood glucose levels above 350 mg/dL. Average body weight was equal in all groups before the induction of diabetes. Four groups were studied: non-diabetic wild type (WT) (*n* = 6), diabetic WT (*n* = 6), non-diabetic NOX2^−/−^ (*n* = 6), diabetic NOX2^−/−^ (*n* = 6). The mice were euthanized after 16 weeks of diabetes. Retinal inflammation and vascular dysfunction are evident at this time but vascular drop-out has not yet begun [26].

### 2.2. Endothelial Cell Culture

Primary cultures of bovine retinal endothelial cells (BREC) at passage 5–8 were used following the method established in our laboratory [27]. Cells were maintained in M199 medium (Cell Systems, Kirkland, WA, USA) with 5 mM glucose, 10% fetal bovine serum (FBS), 100 U/mL penicillin, and 100 mg/mL streptomycin in an atmosphere of 95% air and 5% CO_2_ in a humidified 37 °C incubator. 

### 2.3. High Glucose or Hydrogen Peroxide Treatment of Endothelial Cells

At 80% confluence, the cultures were switched to serum-free medium containing 0.1% bovine serum albumin (BSA) and treated with the NOX2-specific blocking peptide gp91ds-tat (YGRKKRRQRRRCSTRIRRQL-NH_2_) [28]. A scrambled control peptide (SC) (YGRKKRRQRRRCLRITRQSR-NH_2_) was used as a control. Both peptides were used at a concentration of 2 µM (Anaspec, San Jose, CA, USA). After 2 h, the cultures were switched to 25 mM glucose (HG) or 5 mM glucose (NG) media along gp91ds-tat or SC peptide and maintained for 72 h. Other cultures were treated with H_2_O_2_ (5, 15, 25, 50 µM, Sigma-Aldrich, St. Louis, MO, USA) for different times. To assess the contribution of arginase activity, some cultures were treated with or without the arginase inhibitor ABH (2(S)-amino-6-boronohexanoic acid, 100 µM) [29,30]. The involvement of NOS activity was evaluated by treating other cultures with or without l-NAME (l-NG-nitroarginine methyl ester, 100 µM, Sigma-Aldrich, St. Louis, MO, USA) and/or the NO donor SNAP (S-nitroso-N-acetyl-D, l-penicillamine, 10 µM, Sigma-Aldrich, St. Louis, MO, USA).

### 2.4. Nitric Oxide Assays

The NO fluorescent indicator 4-5-diaminofluorescein diacetate (DAF-2 DA, Calbiochem Laboratories) was used to assess production of NO in retinal tissue sections and cultured BRECs. In the presence of oxygen, DAF-2 DA reacts with NO to yield the highly fluorescent triazolofluorescein which is monitored using excitation and emission wavelengths of 485 and 538 nm, respectively. Unfixed frozen retinal sections and fresh BRECs from each group were reacted with DAF-2 DA (10 µM) at 37 °C for 15 min in the dark. To control for NOS specificity, other sets were pre-incubated with l-NAME (1 mM) and then reacted with DAF-2 DA. Next, the slides or cultures were washed with HEPES buffer (10 mM), covered, and the images were collected using the Axioplan2 microscope. Fluorescence intensity was measured using Metamorphic Image System. NO release by the cultured cells was quantified by using a Nitric Oxide Analyzer (Sievers, Boulder, CO, USA) for analysis of the cell-conditioned media. Samples containing NO_2_ were injected in glacial acetic acid containing sodium iodide. NO_2_ is quantitatively reduced to NO under these conditions and is quantified by a chemiluminescence detector after reaction with ozone in the NO analyzer.

### 2.5. Superoxide Assay

To assay superoxide production retinal tissue sections, all groups were reacted with the oxidative fluorescent dye dihydroethidium (DHE, Calbiochem Laboratories, San Diego, CA, USA) following the method described in our previous studies [21]. DHE images from freshly frozen serial sections were obtained using a fluorescence microscope. To confirm the reaction specificity for superoxide, samples were preincubated and maintained in cell permeable superoxide dismutase (PEG-SOD) for 30 min. For studies in cultured cells, cells in 6-well plates were reacted with DHE using the same method. Cells were pretreated with a gp91ds-tat or with the scrambled peptide control (SC) as described above. Images were analyzed for reaction intensity using the Metamorphic Image System (Molecular Devices Corp., Downingtown, PA, USA). 

### 2.6. Arginase Activity Assay

Arginase activity was assayed by measuring urea production from l-arginine as previously described [31]. Briefly, retina samples or BREC were homogenized in ice-cold lysis buffer (1:4 w/v 50 mM Tris-HCl, 100 µM EDTA, and EGTA pH 7.5) containing protease and phosphatase inhibitors. Homogenates were sonicated and centrifuged at 14,000× *g* for 20 min at 4 °C and supernatants were collected for enzyme assay. Twenty-five microliters of supernatants in triplicate were added to 25 µL of Tris-HCl 121 (50 mM, pH 7.5) containing MnCl_2_ (10 mM) and the mixture was activated by heating for 10 min at 55–60 °C. 

### 2.7. Senescence Associated β-Galactosidase (SA-β-Gal) Activity Assay

Mammalian cells express lysosomal β-galactosidase activity at pH of 4.0. Cells in a state of replicative senescence exhibit SA-β-gal activity at pH of 6. Incubation with the chromogenic substrate 5-bromo-4-chloro-3-indolyl-d-galactopyranoside (X-gal) at pH of 6.0 produces intense blue coloration in senescent cells [32]. For SA-β-gal assay retinal sections or BREC cultures were processed following the instructions provided by the manufacturer (Bio Vision laboratory, Milpitas, CA, USA) and incubated overnight at 37 °C with X-gal chromogenic substrate at pH 6.0. The samples were viewed by phase contrast on an Axioplan2 microscope and serial pictures were taken at 200× magnification. The number of SA-β-gal positive cells per mm^2^ was quantified in the tissue culture samples using ImageJ software. The investigators were blinded to the treatment conditions.

### 2.8. Western Blot

BRECs and retinas were homogenized in a modified RIPA buffer (20 mM Tris-HCl [pH 7.4], 2.5 mM EDTA, 50 mM NaF, 10 mM Na_4_P_2_O_7_, 1% Triton X-100, 0.1% sodium dodecyl sulfate, 1% sodium deoxycholate, and 1 mM phenyl methyl sulfonyl fluoride) as described previously [21]. Equal amounts of protein samples were separated by 12% or 8% sodium dodecyl sulfate polyacrylamide gel electrophoresis, transferred to nitrocellulose membrane, and reacted for 24 h with anti-arginase 1 (Goat anti-mouse 1:5000, Abcam, Cambridge, MA, USA), anti-p16(INK4a) (Rabbit polyclonal antibody, 1:500, Santa Cruz Biotechnology, Dallas, TX, USA), anti-NOX2 (Rabbit polyclonal antibody at 1:3000, EMD Millipore Billerica, MA, USA), followed by horseradish peroxidase-linked secondary antibody (GE Healthcare Bio-Science Corp., Piscataway, NJ, USA) to detect immunoreactive proteins. Data were quantified by densitometry using NIH ImageJ and normalized to loading control. Equal loading was verified by stripping the membranes and probing them with a mouse antibody against β-actin (1:5000, Sigma-Aldrich, St. Louis, MO, USA). All antibodies were diluted in 2% BSA.

### 2.9. Statistical Analysis

Group differences were evaluated by using one-way analysis of variance (ANOVA) and post-hoc test. Results were considered significant at *p* < 0.05. Data are presented as the mean *±* SE.

## 3. Results 

### 3.1. Effects of NOX2 Deletion on Arginase Expression/Activity and NO Formation

**Figure 1:**
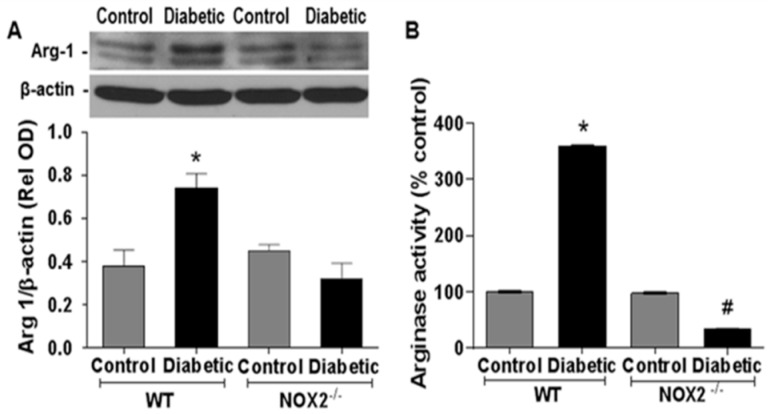
NADPH oxidase 2 (NOX2) deletion prevents diabetes-induced increases in arginase 1 expression and activity. Effects of NOX2 deletion on expression of arginase l (Arg1) protein (**A**) and arginase activity (**B**) in diabetic and control retinas. * *p* < 0.05 vs. control, # *p* < 0.05 vs. WT diabetic, Values are mean ± SEM, *n* = 4–5**.**

**Figure 2:**
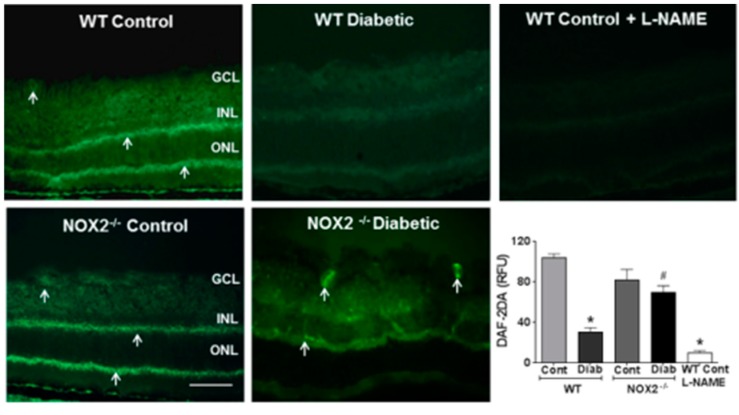
NOX2 deletion prevents diabetes-induced decreases in retinal nitric oxide (NO). Representative images and quantitation showing effects of NOX2 deletion on fluorescence for the NO indicator DAF-2DA (arrows) in diabetic and control retinas. Quantitative analysis showed significant reduction of NO levels in samples from WT diabetic retinas as compared to control retinas. NO was restored by deletion of NOX2. Pretreatment with the NOS inhibitor L-NAME significantly decreased NO in WT retinas. * *p* < 0.05 vs. WT control, # *p* < 0.05 vs. WT diabetic. Values are mean ± SEM, *n* = 4–5. Scale bar = 50 μm.

We determined the involvement of NOX2 in diabetes-induced increases in retinal arginase expression/activity and NO formation by experiments using wild type and NOX2^−/−^ diabetic mice. These experiments showed that diabetes-induced increases in arginase 1 expression and arginase activity were completely blocked in diabetic NOX2**^−/−^** mice, indicating that NOX2 expression has a key role in this process (Figure 1A,B). Further analyses using DAF-2DA imaging showed that the diabetes-induced decreases in NO formation were markedly abrogated in retinas from the NOX2^−/−^ diabetic mice. NO formation was largely protected and remained similar to that in the non-diabetic controls (Figure 2). Treatment of the sections with the NOS inhibitor l-NAME markedly suppressed the DAF-2DA reaction, indicating the specificity of the reaction for NO. Taken together, these results suggest that NOX2 activation is involved in the diabetes-induced upregulation of arginase expression/activity and down-regulation of NO production.

Studies using NOX2 Western blotting and DHE imaging support the role of NOX2 expression and NOX2-derived ROS in this process. Western blot analysis showed a significant increase in NOX2 expression in WT diabetic mice as compared with the non-diabetic controls (Supplemental Figure S1). DHE imaging showed a substantial increase in ROS in WT diabetic mice as compared with the non-diabetic control mice (Supplemental Figure S2). This increase in DHE reactivity was completely prevented in diabetic NOX2^−/−^ mice. Treatment with PEG-SOD also blocked DHE reaction, indicating its specificity for superoxide. These results demonstrate the involvement of NOX2-derived ROS formation in the diabetes-induced increases in arginase expression/activity and decreases in NO formation. 

### 3.2. Effects of NOX2 Deletion/Blockade on Diabetes- or High Glucose-Induced Increases in SA-β-Gal Activity

**Figure 3:**
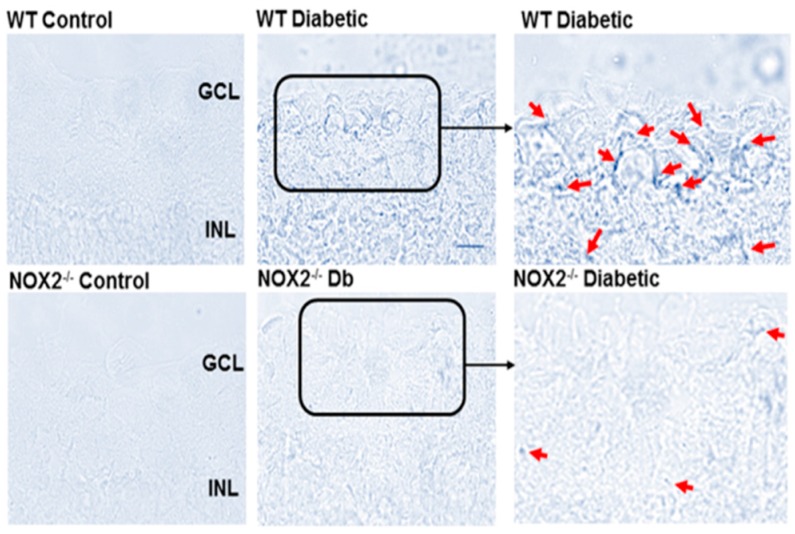
NOX2 deletion limits diabetes-induced increases in SA-β-gal activity. Representative images showing effects of diabetes on SA-β-gal activity in the inner retina. Retinas of WT diabetic mice showed numerous SA-β-gal positive cells (red arrows) in the inner retina. The SA-β-gal signal is largely absent in retinas from the NOX2^−/−^ diabetic mice. Scale bar = 50 μm.

**Figure 4:**
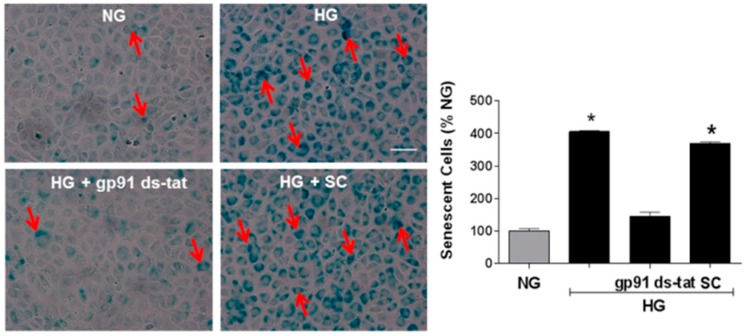
NOX2 blockade limits high glucose-induced increases in SA-β-gal activity. Representative images and statistical analysis showing effects of high glucose on SA-β-gal activity in retinal endothelial cells. Incubation of the cultures in high glucose (HG) media markedly increased the number of SA-β-gal-positive cells (arrows) as compared with the normal glucose (NG) controls. This was prevented by treatment with the NOX2-blocking peptide gp91ds-tat. Treatment with the scrambled control (SC) peptide did not alter the high glucose effect. * *p* < 0.0001 vs. NG, *n* = 6. Scale bar = 20 µm.

**Figure 5:**
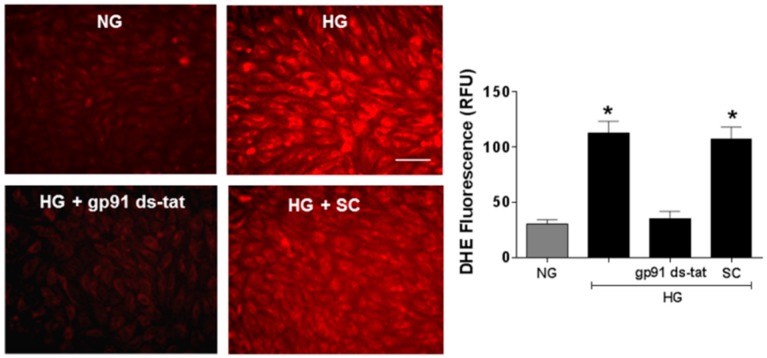
NOX2 blockade prevents high glucose-induced increases in oxidative stress. Representative images and statistical analysis of dihydroethidium (DHE) imaging showing effects of high glucose on reactive oxygen species (ROS) formation in endothelial cells. Treatment with high glucose (HG) markedly increased the DHE fluorescence as compared with the normal glucose controls (NG). Treatment with the NOX2-blocking peptide gp91ds-tat but not the scrambled control (SC) peptide blocked the HG-induced increase in ROS. * *p* < 0.05, *n* = 10. Scale bar = 20 µm.

Analysis of SA-β-gal activity at pH 6.0 in retinal sections showed a prominent increase in SA-β-gal activity in the inner retinas of WT diabetic mice (Figure 3). Based on their location in the inner retina, it appears that the SA-β-gal-positive senescent cells are localized to the area of the retinal vessels within the ganglion cell layer. However, the phase contrast images do not allow conclusive identification of the vascular endothelium and other cell types may also be involved. Thus, we performed further studies using an in vitro model system to assess the effects of high glucose (HG) treatment on SA-β-gal activity in retinal ECs in relation to NOX2 NADPH oxidase activity. We found that HG treatment caused a marked increase in the SA-β-gal reaction in the retinal ECs (Figure 4) along with a prominent increase in ROS formation as shown by DHE imaging (Figure 5). Both effects were almost completely prevented by treatment of the cultures with the NOX2-specific blocking peptide gp91 ds-tat [28], whereas treatment with the scrambled control peptide had no effect.

### 3.3. Effects of NOX2 Blockade on High Glucose-Induced Arginase Expression and Activity and NO Formation

**Figure 6:**
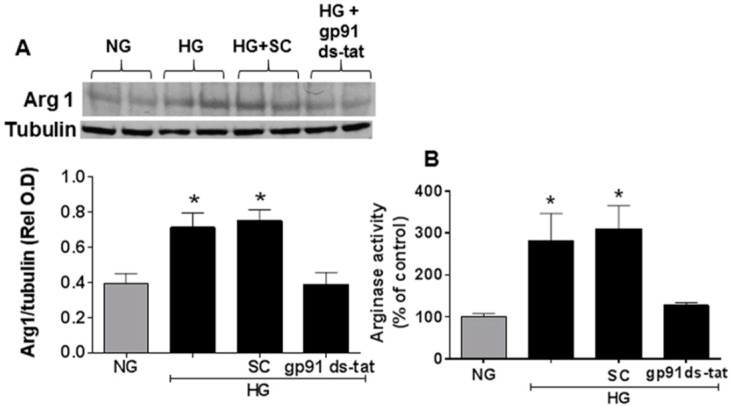
NOX2 blockade prevents high glucose-induced increases in arginase 1 expression and arginase activity in retinal endothelial cells. Western blot and quantitation showing effects of the NOX2-blocking peptide gp91ds-tat on expression of arginase l (Arg1) protein (**A**) and arginase activity (**B**) in endothelial cells treated with high glucose (HG). HG substantially increased arginase 1 expression and arginase activity as compared to the normal glucose (NG) controls. These increases were blocked by bp91ds-tat but were not altered by the scrambled control (SC) peptide. * *p* < 0.05, *n* = 3–4.

**Figure 7:**
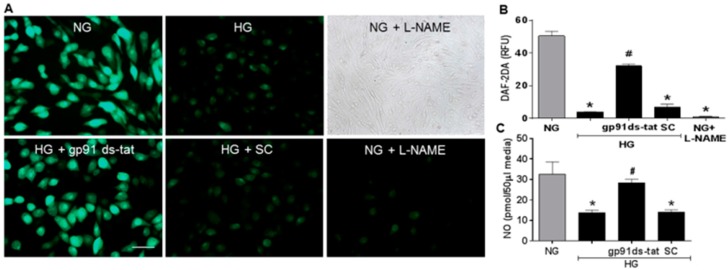
NOX2 blockade prevents high glucose-induced decreases in endothelial cell nitric oxide. Representative images (**A**) and quantitation (**B**) showing effects of NOX2 blockade on fluorescence for the NO indicator DAF-2DA in endothelial cells maintained in high glucose (HG) or normal glucose (NG) media. Quantitative analysis showed significant reduction of NO levels in samples from HG-treated cells as compared to NG controls. NO was preserved in HG cultures treated with the NOX2-blocking peptide gp91ds-tat but not in cultures treated with the scrambled control (SC) peptide. Treatment with the NOS inhibitor L-NAME significantly decreased NO in the NG cultures but did not alter cell density as shown by phase contrast image. * *p* < 0.0001 vs. NG, # *p* < 0.05 vs. HG, *n* = 6. Analysis of NO accumulation in the conditioned media (C) confirmed a decrease in NO release in the HG cultures that was blocked by gp91ds-tat, but not by the scrambled control (SC). * *p* < 0.05 vs. NG, # *p* < 0.05 vs. HG, *n* = 4–6. Scale bar = 20 µm.

Studies using the same in vitro model system confirmed the suggested association of NOX2 activation and accelerated EC senescence with elevation of arginase expression and activity and depression of NO formation. Western blot and urea formation assays showed that the high glucose treatment of the retinal ECs caused significant increases in arginase 1 protein levels as well as arginase activity (Figure 6). Analyses using DAF-2 DA imaging and chemiluminescence showed marked decreases in NO formation as compared with the control cells maintained in normal glucose media (Figure 7). All of these high glucose-induced alterations were prevented by treatment of the cultures with the NOX2-blocking peptide gp91 ds-tat, whereas treatment with the scrambled peptide control did not alter the high glucose-induced changes (Figure 6 and Figure 7).

### 3.4. Involvement of Arginase and NOS Activity in ROS-Induced EC Senescence 

**Figure 8:**
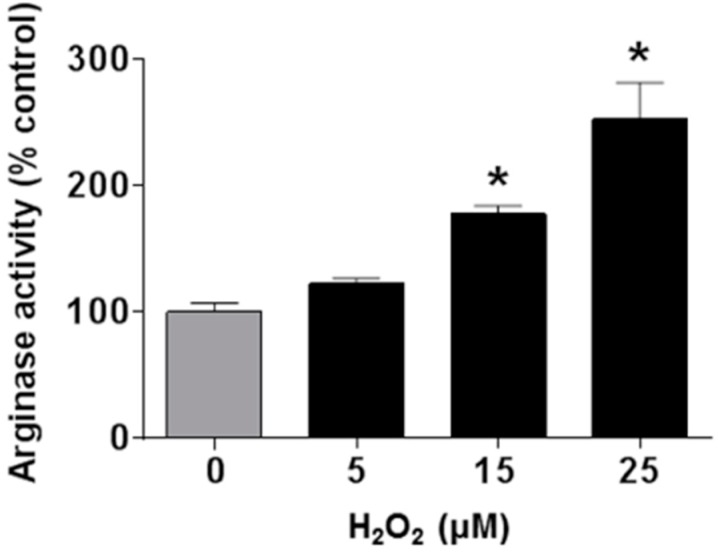
Hydrogen peroxide induces an increase in arginase activity. Treatment of retinal endothelial cells with hydrogen peroxide induced a dose-dependent increase in arginase activity as compared to control media. * *p* < 0.05 vs. control, *n* = 3–4.

**Figure 9:**
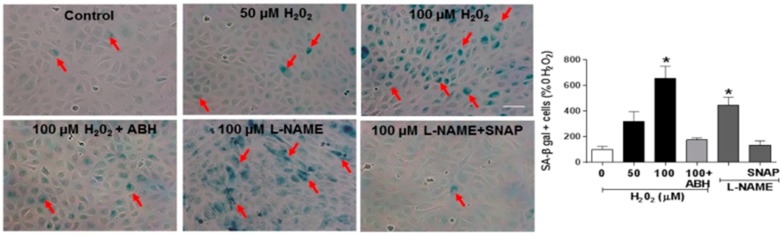
Hydrogen peroxide induces an increase in SA-β-gal activity. Treatment of retinal endothelial cells with hydrogen peroxide or the NOS inhibitor l-NG-nitroarginine methyl ester (l-NAME) induced a significant increase in SA-β-gal activity (arrows). Treatment with the arginase inhibitor ABH blocked the hydrogen peroxide effect and treatment with the NO donor SNAP blocked the L-NAME effect. * *p* < 0.05 vs. control, *n* = 17. Scale bar = 30 µm.

**Figure 10:**
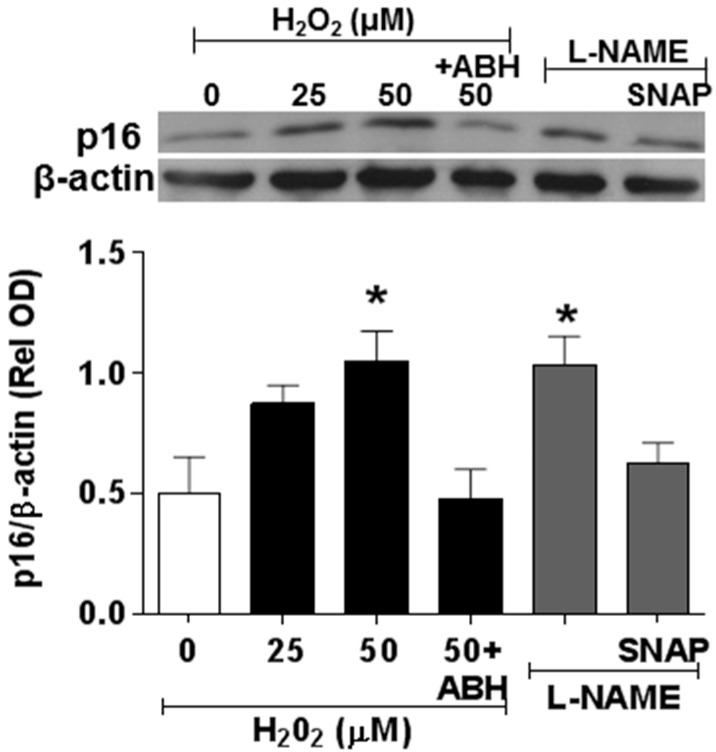
Hydrogen peroxide induces an increase in the cyclin-dependent kinase inhibitor p16INK4a. Western blot and quantitation showing effects of treatment with hydrogen peroxide or l-NAME in increasing expression of p16INK4a protein. These effects were blocked by the arginase inhibitor ABH (100 µM) or the NO donor SNAP (10 µM), * *p* < 0.05 vs. control, *n* = 3–4.

We next examined the impact of ROS-induced upregulation of arginase activity and down-regulation of NO formation in premature EC senescence by studies using ECs treated with hydrogen peroxide or the NOS inhibitor l-NAME. These experiments showed that hydrogen peroxide treatment caused a dose-dependent increase in arginase activity (Figure 8) along with parallel increases in SA-β-gal activity (Figure 9) and increased expression of the senescence marker and cyclin-dependent kinase inhibitor p16(INK4a) (Figure 10). Each of these effects of hydrogen peroxide was blocked in cultures treated with the arginase inhibitor ABH or NO donor SNAP (Figure 9 and Figure 10). By contrast, treatment with the NOS inhibitor l-NAME caused significant increases in numbers of SA-β-gal-positive cells along with increased expression of p16(INK4a). 

## 4. Discussion

In this study, we investigated the effects of diabetes-induced increases in NOX2-NADPH oxidase expression on premature EC senescence in relation to arginase expression/activity and NO formation. We found that diabetes- or high glucose-induced increases in NOX2/NADPH-generated ROS induce premature EC senescence by a mechanism involving increases in arginase 1 expression and activity and decreases in NO bioavailability. Inhibition of NOX2 activity in EC or deletion of NOX2 in mice importantly prevented premature EC senescence by limiting the diabetes- or high glucose-induced increases in arginase expression and activity.

The role of oxidative stress in diabetic complications, including retinal vascular injury and dysfunction, is well established [6,33]. An association between diabetes and premature vascular endothelial cell senescence has also been well documented [34]. However, this study is the first we know of to show a link between NOX2, arginase 1 and diabetes-induced retinal endothelial cell senescence. Using an in vitro model, we found that high glucose-induced increases in endothelial senescence, ROS formation, and elevated arginase expression/activity were all prevented by the NOX2-blocking peptide gp91sd-tat (Figure 4, Figure 5 and Figure 6). Furthermore, diabetes- or oxidative stress-induced decreases in NO formation were prevented by deletion of NOX2 in vivo (Figure 2) or blockade of NOX2 in vitro (Figure 7), suggesting a causal link between NOX2-induced arginase activation and suppression of NO bioavailability. The association between NOX2-induced increases in oxidative stress and endothelial dysfunction in diabetes has been well established [35]. Reactive oxygen species are known to increase expression and activity of endothelial arginase [9,24,25,36]. To reproduce the ROS-induced endothelial cell damage/dysfunction associated with diabetes and high glucose conditions, we treated the retinal endothelial cells with hydrogen peroxide. This treatment caused a dose-dependent increase in arginase activity that was correlated with increases in SA-β-gal-positive endothelial cells and expression of p16(INK4a) [37] (Figure 8, Figure 9 and Figure 10). Senescent cells often express p16INK4a, a cyclin-dependent kinase inhibitor, tumor suppressor and biomarker of aging [38]. Cellular senescence was also increased by treatment of the cells with the NOS inhibitor l-NAME. The hydrogen peroxide or l-NAME-induced increases in cellular senescence were blocked by co-treatment of the cultures with the arginase inhibitor ABH or the NO donor SNAP (Figure 9 and Figure 10), suggesting a mechanism involving arginase-induced decreases in NO bioavailability. ABH is highly specific for arginase activity; it inhibits both arginase isoforms and does not alter NOS activity [29,30]. 

The clinical significance of this study is reflected in the beneficial effects of inhibition of NOX2 or arginase activity in preventing oxidative stress and preserving NO bioavailability, which prevents premature cellular senescence. Previous studies have shown that NO can S-nitrosylate NOX2 and thus inhibit its activity [39]. In addition, the NOX1, NOX3, and NOX5 isoforms also are sensitive to NO-dependent inhibition via s-nitrosylation [40]. In our study, treatment with the arginase inhibitor produced results similar to those with the NO donor, but results opposite to those observed with l-NAME, indicating that arginase inhibition represents a mechanism to restore endogenous synthesis of NO. Our analysis of this point led us to consider senescence as a reversible process. If cells under stress are able to produce sufficient NO, they may revert to normal physiological function. Testing this possibility, however, would require more study. The cascade of events caused by elevated arginase activity includes lowering NO levels, which will lead to decreases in guanylate cyclase activity and therefore reduction in cGMP production. cGMP is implicated in the process of senescence [41] and also is a regulator of ion channel conductance, glycogenolysis, cellular apoptosis and vascular tone [42]. It is also a secondary messenger in retinal photo-transduction [43], a process by which light is converted into electrical signals in the photoreceptors. Our data suggest that diabetes-induced activation of the NOX2/arginase pathway is fundamentally involved in reducing NO and cGMP levels in the retinal vascular endothelial cells. Further study is needed to assess the role of this pathway in diabetes-induced alterations in retinal photo-transduction. Further study is also needed to extend these results to models of type 2 diabetes. However, we believe that similar mechanisms are operative given that type 2 diabetic subjects experience hyperglycemia and vascular dysfunctions similar to those seen in type 1 diabetes.

**Figure 11:**
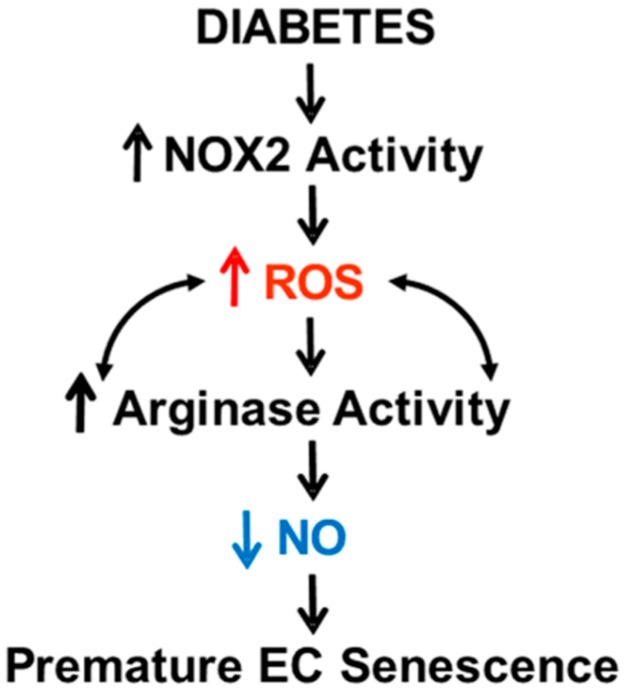
Hypothesis: Diabetes-induced activation of NOX2/NADPH oxidase promotes the development of premature endothelial cell senescence by a mechanism involving ROS-induced activation of arginase which amplifies ROS and decreases bioavailable NO.

Our studies support the hypothesis that diabetes-induced EC senescence involves sequential events initiated by NOX2 activation and production of ROS leading to increased endothelial arginase expression and activity, reduced availability of l-arginine to NOS and decreased production of NO (Figure 11). This process can also uncouple NOS leading to increased production of superoxide, its reaction with NO to produce peroxynitrite, which not only further decreases NO bioavailability, but also can increase and maintain arginase expression in a feed-forward manner [9,24]. It also is important to recognize that other sources of ROS elevated by diabetes may also increase and maintain elevated arginase expression and activity. While we did not examine the effects of diabetes and oxidative stress on arginase 1 mRNA expression in the present study, studies in other models suggest that arginase activity in endothelial cells is proportional to the amount of arginase protein, which, in turn, is determined primarily by transcription of the arginase genes [25].

## Supplementary Materials

The following are available online at www.mdpi.com/2076-3921/6/2/43/s1. Figure S1: Diabetes induces an increase in NOX2 expression. Western blot and quantitation showing effects of diabetes in increasing NOX2 expression. * *p* < 0.05, *n* = 4. Figure S2: NOX2 deletion prevents diabetes-induced decreases in retinal oxidative stress. Representative DHE images and quantitation showing effects of diabetes on ROS formation in retinal tissue. Diabetes markedly increased the DHE fluorescence as compared with the non-diabetic controls. This increase was completely blocked by NOX2 deletion. The DHE increase was completely blocked by pre-incubation with SOD, demonstrating specificity for superoxide. * *p* < 0.05, *n* = 9. Scale bar = 20 µm.

## Abbreviations

ROS: reactive oxygen species, NO: nitric oxide, NOS: nitric oxide synthase, eNOS: endothelial nitric oxide synthase, EC: endothelial cells, BH_4_: tetrahydrobiopterin, HUVEC: human umbilical vein endothelial cells, NOX2: NADPH oxidase 2, VEGF: vascular endothelial growth factor, ICAM1: intercellular adhesion molecule 1, STZ: streptozotocin.
