# Supplementary material for: NOX2-Induced Activation of Arginase and Diabetes-Induced Retinal Endothelial Cell Senescence
Source: Antioxidants (Basel). 2017 Jun 15;6(2):43. doi: 10.3390/antiox6020043 (PMC5488023; doi:10.3390/antiox6020043)
Supplement: Supplementary file 1 [file antioxidants-06-00043-s001.pdf]

antioxidants

Supplementary Materials

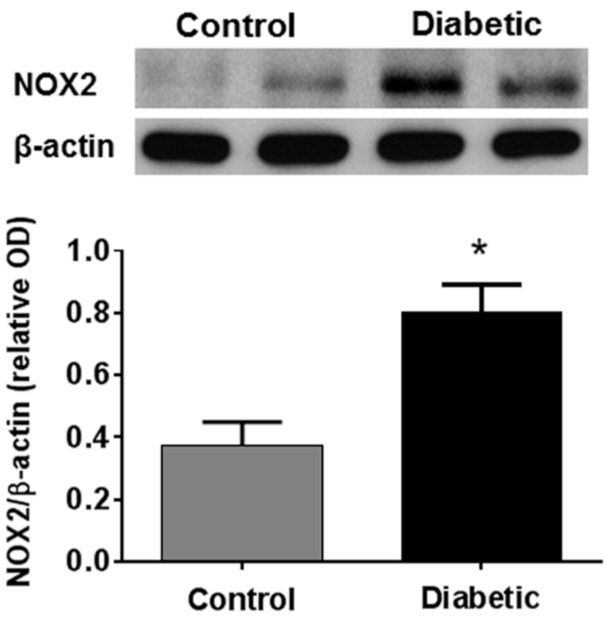

Figure S1. Diabetes induces an increase in NOX2 expression. Western blot and quantitation showing effects of diabetes in increasing NOX2 expression. * $p < 0.05$, $n = 4$.

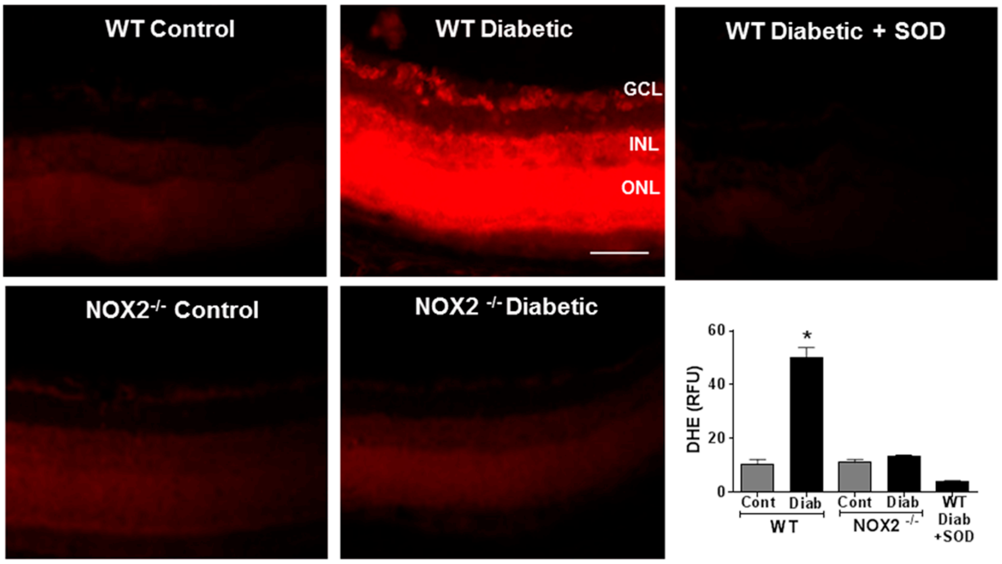

Figure S2. NOX2 deletion prevents diabetes-induced decreases in retinal oxidative stress. Representative DHE images and quantitation showing effects of diabetes on ROS formation in retinal tissue. Diabetes markedly increased the DHE fluorescence as compared with the non-diabetic controls. This increase was completely blocked by NOX2 deletion. The DHE increase was completely blocked by pre-incubation with SOD, demonstrating specificity for superoxide. * $p < 0.05$, $n = 9$. Scale bar = 20 μm.
